# Supplementary material for: Artificial intelligence empowering museum space layout design: Insights from China
Source: PLoS One. 2024 Nov 7;19(11):e0310594. doi: 10.1371/journal.pone.0310594 (PMC11542801; doi:10.1371/journal.pone.0310594)
Supplement: S1 File — (DOCX) [file pone.0310594.s001.docx]

# S1. Machine Learning Environment Configuration

The operating system is Windows 11 (X64), the CUDA version is 11.5, the deep learning framework is PyTorch (1.13.0), and the graphics card and processor are a GeForce GTX 3070 (16 G) and an AMD Ryzen 9 5900HX (3.30 GHz), respectively.
