# Supplementary material for: Artificial intelligence empowering museum space layout design: Insights from China
Source: PLoS One. 2024 Nov 7;19(11):e0310594. doi: 10.1371/journal.pone.0310594 (PMC11542801; doi:10.1371/journal.pone.0310594)
Supplement: S2 File — (DOCX) [file pone.0310594.s002.docx]

# S2. Research Materials (Museum Floor Plan) Source Statistics

The list of Chinese museums involved in this study is as follows, and they are divided into five categories: art, history, science, nature, and comprehensive.

| No. | Museum Type | Project Name | Location (address) | Year of construction | Area (m^2^) |
| --- | --- | --- | --- | --- | --- |
| 1 | Science Museum | Qinghai Science and Technology Museum | Kepu Road, Chengxi District, Xining City, Qinghai Province | 1984 | 33179 |
| 2 |  | The Museum of Chinese Medicine at the Beijing University of Chinese Medicine | North Third Ring Road East, Beijing | 1990 | 3160 |
| 3 |  | Liaoning Paleontological Museum | No. 253, Huanghe North Street, Huanggu District, Shenyang City, Liaoning Province | 2011 | 15000 |
| 4 |  | Qiantang River Museum | The intersection of Beijing-Hangzhou Grand Canal and Qiantang River in Hangzhou City, Zhejiang Province | 2019 | 1100 |
| 5 | Nature Museum | China Agricultural Museum | No.16, East Third Ring North Road, Chaoyang District, Beijing | 1983 | 12400 |
| 6 |  | China Geological Museum Yantai Pavilion and Yantai Natural History Museum | No. 2001, Binhai Middle Road, High-tech Zone, Yantai City, Shandong Province | 2006 | 26300 |
| 7 |  | China East China Sea Crystal Museum | No. 1, Zhonghua Road, Donghai County, Lianyungang City, Jiangsu Province | 2012 | 29000 |
| 8 |  | Thousand Island Lake Natural Museum | Within Thousand Island Lake, Chun'an County, Hangzhou, Zhejiang Province | 2018 | 6000 |
| 9 | Historical Museum | The Museum of Dr. Sun Yat-sen | Cuiheng Village, Zhongshan City, Guangdong Province | 1956 | 8400 |
| 10 |  | Hunan Province Xiangxi Tujia and Miao Autonomous Prefecture Museum | Furonggang Road, Jishou City, Xiangxi Tujia and Miao Autonomous Prefecture, Hunan Province | 1957 | 37891 |
| 11 |  | Su Dongpo Memorial Hall | Fengshan Park, Huicheng District, Huizhou City, Guangdong Province | 1982 | 2500 |
| 12 |  | Zhuzhou City Museum | No. 205, Zhujiang North Road, Tianyuan District, Zhuzhou City, Hunan Province | 1984 | 20516 |
| 13 |  | Nanyue Sages Hall | No. 233, Huifu West Road, Yuexiu District, Guangzhou City, Guangdong Province | 1985 | 6430 |
| 14 |  | Su Dongpo Memorial Hall | No. 2, Nanshan Road, Xihu District, Hangzhou City, Zhejiang Province | 1988 | 550 |
| 15 |  | China Numismatic Museum | No. 17, Xijiaomin Lane, Xicheng District, Beijing | 1990 | 1800 |
| 16 |  | Hemudu Ruins Museum | Lushansi Village, Hemudu Town, Yuyao City, Ningbo City, Zhejiang Province | 1991 | 3000 |
| 17 |  | Anqing Revolutionary Cultural Relics Exhibition Hall (Huangzhen Life and Deeds Exhibition Hall) | No. 102, Linghu South Road, Yingjiang District, Anqing City, Anhui Province | 1993 | 5800 |
| 18 |  | China Shipbuilding Culture Museum | No. 7, Zhaozhong Road, Mawei District, Fuzhou City, Fujian Province | 1997 | 4100 |
| 19 |  | Opium War Museum | No. 88 Jiefang Road, Humen Town, Dongguan City, Guangdong Province | 1998 | 10000 |
| 20 |  | Shenzhen Museum History and Folklore Museum | Area A, Civic Center, Fuzhong Road, Futian District, Shenzhen City, Guangdong Province | 1998 | 7045 |
| 21 |  | Museum of Hakka Family Tree | Hakka Culture Center, Longxiang Avenue, Shanghang County, Fujian Province | 2000 | 30000 |
| 22 |  | East China Sea Fleet Military History Museum | Bank of Dongqian Lake, Yinzhou District, Ningbo City, Zhejiang Province | 2003 | 3015 |
| 23 |  | Jinggangshan Revolutionary Museum (New Building) | Red Army South Road, Ciping, Jinggangshan City, Jiangxi Province | 2007 | 8436 |
| 24 |  | Ningxia Transportation Museum | No. 175, Beijing Middle Road, Jinfeng District, Yinchuan City, Ningxia Hui Autonomous Region | 2008 | 38887 |
| 25 |  | Xinhai Revolution Memorial Hall | Changzhou Island, Huangpu District, Guangzhou City, Guangdong Province | 2009 | 58000 |
| 26 |  | Anhui Fame Hall | No. 303, Yungu Road, Binhu New District, Hefei City, Anhui Province | 2009 | 38000 |
| 27 |  | Nanjing Anti-Japanese Aviation Martyrs Memorial Hall | North foot of Purple Mountain, Xuanwu District, Nanjing City, Jiangsu Province | 2009 | 2200 |
| 28 |  | China Railway Museum (Zhengyang Gate) | North side of No. 1 Courtyard, Jiuxianqiao North Road, Chaoyang District, Beijing | 2010 | 4000 |
| 29 |  | China Bronzeware Museum (New Building) | Shigushan, Binhe South Road, Baoji City, Shaanxi Province | 2010 | 2250 |
| 30 |  | Nanjing Dabaoen Temple Ruins Museum | No. 1, Yuhua Road, Qinhuai District, Nanjing City, Jiangsu Province | 2012 | 60600 |
| 31 |  | Tashan Blockade Memorial Hall | Tashan Township, Lianshan District, Huludao City, Liaoning Province | 2013 | 3000 |
| 32 |  | The Oriental Metropolitan Museum | No. 302, Changjiang Road, Xuanwu District, Nanjing City, Jiangsu Province | 2014 | 23000 |
| 33 |  | Helv Relics Museum | Auxiliary Road around Taihu Lake Highway in Binhu District, Wuxi City, Jiangsu Province | 2014 | 27000 |
| 34 |  | Lingnan Finance Museum | Lujiang Academy, No. 29, Liushuijing, Education Road, Yuexiu District, Guangzhou City, Guangdong Province | 2018 | 2300 |
| 35 |  | Panlong City Ruins Museum | No. 1, Panlong Avenue, Panlongcheng Economic Development Zone, Huangpi, Wuhan City, Hubei Province | 2016 | 16300 |
| 36 |  | Shanxi Bronze Museum | Changfeng Business District, Taiyuan, Shanxi Province | 2019 | 11000 |
| 37 |  | Zhongshan City Museum (New Building) | No. 197, Sunwen Middle Road, Zhongshan City, Guangdong Province | 2022 | 24832 |
| 38 | Comprehensive museum | Zhejiang Provincial Museum | No. 25 Gushan Road, Hangzhou, Zhejiang Province | 1929 | 7360 |
| 39 |  | The Ningxia Hui Autonomous Region Museum | No. 6, People's Square East Road, Yinchuan City, Ningxia Hui Autonomous Region | 1959 | 30285 |
| 40 |  | Foshan Municipal Museum | No. 21, Zumiao Road, Foshan City, Guangdong Province | 1959 | 25500 |
| 41 |  | Guangzhou Luxun Museum | No. 215, Wenming Road, Yuexiu District, Guangzhou City, Guangdong Province | 1959 | 2500 |
| 42 |  | National Museum of Ganzhou | No. 15, Xingguo Road, Zhanggong District, Ganzhou City, Jiangxi Province | 1960 | 28500 |
| 43 |  | Longyan Municipal Museum | No. 2, Longyan Avenue Middle, Xinluo District, Longyan City, Fujian Province | 1975 | 20000 |
| 44 |  | Hainan Museum | No. 76, Guoxing Avenue, Qiongshan District, Haikou City, Hainan Province | 1984 | 43000 |
| 45 |  | Lishui City Museum | No. 30, Dayou Street, Lishui City, Zhejiang Province | 1984 | 13800 |
| 46 |  | Taihu County Museum | 400 meters west of Taihu Toll Station on Huyu Expressway, Taihu County, Anqing City, Anhui Province | 1986 | 4000 |
| 47 |  | Zhangzhou Museum | No. 230, Yingbin Avenue, Longwen District, Zhangzhou City, Fujian Province | 1988 | 4600 |
| 48 |  | Henan Museum | No. 8, Nongong Road, Jinshui District, Zhengzhou City, Henan Province | 1997 | 55000 |
| 49 |  | Shanxi Museum | No. 13, North Section of Binhe West Road, Taiyuan City, Shanxi Province | 2001 | 52000 |
| 50 |  | Nantong City Museum | No. 1, Huancheng South Road, Chongchuan District, Nantong City, Jiangsu Province | 2005 | 10000 |
| 51 |  | Tianjin Museum | No. 62, Pingjiang Road, Hexi District, Tianjin | 2007 | 64003 |
| 52 |  | Suzhou Museum | 80 meters south of Galaxy Plaza, Yinhe 1st Road, Yongqiao District, Suzhou City, Anhui Province | 2008 | 10000 |
| 53 |  | The Chinese Museum of Women and Children | No. 23, Jianguomen Inner Street, Dongcheng District, Beijing | 2009 | 35000 |
| 54 |  | Susong County Museum | No. 109, Renmin Road, Fuyu Town, Susong County, Anqing City, Anhui Province | 2009 | 528 |
| 55 |  | Wuhu Museum | South of Business and Cultural Center, Chengdong New District, Wuhu City, Anhui Province | 2011 | 22000 |
| 56 |  | Beijing Grand Canal Museum (Capital Museum East Branch) | North side of Urban Green Heart Forest Park, Tongzhou District, Beijing | 2022 | 99700 |
| 57 |  | Sun Yat-sen University Museum (School History Museum) | No. 135, Xingang West Road, Haizhu District, Guangzhou City, Guangdong Province | 2022 | 32000 |
| 58 | Art Museum | Longquan Celadon Museum | Jianchuan Avenue, Chengnan, Longquan City, Lishui City, Zhejiang Province | 1989 | 2000 |
| 59 |  | National Museum of Chinese Writing | No. 656, East Section of Renmin Avenue, Beiguan District, Anyang City, Henan Province | 1999 | 34500 |
| 60 |  | Helan Mountain Rock Art Museum | Jinshan Township, Helan County, Yinchuan City, Ningxia Hui Autonomous Region | 2008 | 4106 |
| 61 |  | China Wood Sculpture Museum | No. 77, Chengnan East Road, Dongyang City, Zhejiang Province | 2008 | 26000 |
| 62 |  | China Wuzhou Kiln Museum | Hanzao Village, Yafan Town, Jinhua City, Zhejiang Province | 2017 | 2700 |
| 63 |  | Guangzhou Museum of Art (New Building) | No. 198, Yizhou Road, Haizhu District, Guangzhou City, Guangdong Province | 2023 | 79947 |
