# Supplementary material for: Artificial intelligence empowering museum space layout design: Insights from China
Source: PLoS One. 2024 Nov 7;19(11):e0310594. doi: 10.1371/journal.pone.0310594 (PMC11542801; doi:10.1371/journal.pone.0310594)
Supplement: S3 File — (DOCX) [file pone.0310594.s003.docx]

# S3. Detailed RGB Values in Label Making

The scenery is blue (R0, G0, B255); a booth is yellow (R255, G255, B0); a square showcase is green (R0, G255, B0); a round showcase is red (R255, G0, B0); an exhibition board is purple (R255, G0, B255); a window is cyan (R0, G255, B255); a display rack is orange (R255, G150, B0); a wall is brown (R125, G0, B0); a background wall is dark green (R0, G125, B0); and the ground is gray (R125, G125, B125).
