# Supplementary material for: Artificial intelligence empowering museum space layout design: Insights from China
Source: PLoS One. 2024 Nov 7;19(11):e0310594. doi: 10.1371/journal.pone.0310594 (PMC11542801; doi:10.1371/journal.pone.0310594)
Supplement: S4 File — (DOCX) [file pone.0310594.s004.docx]

# S4. Detailed Parameter Settings for Model Training

The detailed parameters of the machine learning program during model training in this study are as follows:

| Option | Value | Option | Value |
| --- | --- | --- | --- |
| Batch Size | 1 | Number of Local Blocks | 3 |
| Beta1 for Adam Optimizer | 0.5 | Number of Clusters | 10 |
| Data Type (Bit) | 32 | Number of Downsampling Layers in Encoder | 4 |
| Display Frequency | 100 | Number of Global Downsampling Layers | 4 |
| Number of Features | 3 | Number of Layers in Discriminator | 3 |
| Final Size of Images | 512 | Number of Local Enhancers | 1 |
| Number of Input Channels | 3 | Number of Discriminator Filters | 64 |
| Lambda for Feature Loss | 10 | Number of Encoder Filters | 16 |
| Initial Load Size of Images | 512 | Number of Generator Filters | 64 |
| Learning Rate | 0.0002 | Number of Iterations | 140 |
| Number of Threads | 2 | Number of Iterations to Decay LR | 60 |
| Number of Global Blocks | 9 | Number of Output Channels | 3 |

Source: The author recorded the parameters of the machine learning program when training the model.
