# Supplementary material for: Artificial intelligence empowering museum space layout design: Insights from China
Source: PLoS One. 2024 Nov 7;19(11):e0310594. doi: 10.1371/journal.pone.0310594 (PMC11542801; doi:10.1371/journal.pone.0310594)
Supplement: S5 File — (DOCX) [file pone.0310594.s005.docx]

# S5. Numerical Statistics During the Model 1 Epoch Period

In the Epoch cycle of Model 1, the detailed numerical statistics of G_GAN, G_GAN_Feat, G_VGG, D_real, and D_fake are as follows:

| Epoch | G_GAN | G_GAN_Feat | G_VGG | D_real | D_fake |
| --- | --- | --- | --- | --- | --- |
| 1 | 1.1 | 5.132 | 5.703 | 0.495 | 0.39 |
| 2 | 1.879 | 6.939 | 6.768 | 0.739 | 0.625 |
| 3 | 1.033 | 3.681 | 4.081 | 0.714 | 0.246 |
| 4 | 0.812 | 5.434 | 5.966 | 0.365 | 0.373 |
| 5 | 0.941 | 6.148 | 6.752 | 0.239 | 0.533 |
| 6 | 0.743 | 7.075 | 5.172 | 0.137 | 0.535 |
| 7 | 1.496 | 4.984 | 4.107 | 0.566 | 0.085 |
| 8 | 1.44 | 7.565 | 6.222 | 0.445 | 0.334 |
| 9 | 0.805 | 6.161 | 5.703 | 0.219 | 0.4 |
| 10 | 0.935 | 6.941 | 6.819 | 0.153 | 0.28 |
| 11 | 0.802 | 6.038 | 5.687 | 0.144 | 0.314 |
| 12 | 1.044 | 6.584 | 6.037 | 0.076 | 0.209 |
| 13 | 1.257 | 6.476 | 4.964 | 0.444 | 0.246 |
| 14 | 1.489 | 5.788 | 4.746 | 0.737 | 0.146 |
| 15 | 1.422 | 6.264 | 5.628 | 1.207 | 0.278 |
| 16 | 1.258 | 7.654 | 6.5 | 0.497 | 0.166 |
| 17 | 1.269 | 5.152 | 4.275 | 0.744 | 0.122 |
| 18 | 1.961 | 7.831 | 6.995 | 1.319 | 1.426 |
| 19 | 0.924 | 7.182 | 6.069 | 0.25 | 0.252 |
| 20 | 1.025 | 8.525 | 6.962 | 0.144 | 0.435 |
| 21 | 0.664 | 7.547 | 7.611 | 0.3 | 0.636 |
| 22 | 1.699 | 6.673 | 5.517 | 0.823 | 0.08 |
| 23 | 1.073 | 6.84 | 5.636 | 0.459 | 0.195 |
| 24 | 1.302 | 5.117 | 4.464 | 0.981 | 0.157 |
| 25 | 2.045 | 8.444 | 4.93 | 0.355 | 0.05 |
| 26 | 0.854 | 5.212 | 3.469 | 0.242 | 0.646 |
| 27 | 0.718 | 7.972 | 6.363 | 0.188 | 0.773 |
| 28 | 0.672 | 7.773 | 5.598 | 0.106 | 0.623 |
| 29 | 1.453 | 5.268 | 3.532 | 0.723 | 0.113 |
| 30 | 0.168 | 11.014 | 8.109 | 0.23 | 1.299 |
| 31 | 1.378 | 7.368 | 5.602 | 0.238 | 0.116 |
| 32 | 0.827 | 7.082 | 5.072 | 0.409 | 0.549 |
| 33 | 1.518 | 6.282 | 5.352 | 0.57 | 0.201 |
| 34 | 0.738 | 7.192 | 6.246 | 0.365 | 0.574 |
| 35 | 1.159 | 5.757 | 4.07 | 1.173 | 0.24 |
| 36 | 0.806 | 8.375 | 7.785 | 0.082 | 0.482 |
| 37 | 0.767 | 6.988 | 4.853 | 0.148 | 0.438 |
| 38 | 1.285 | 7.383 | 5.372 | 0.314 | 0.276 |
| 39 | 1.439 | 11.24 | 8.012 | 0.215 | 0.114 |
| 40 | 1.765 | 7.561 | 6.704 | 1.035 | 0.148 |
| 41 | 0.853 | 6.663 | 5.939 | 0.195 | 0.298 |
| 42 | 1.554 | 7.051 | 5.921 | 0.867 | 0.105 |
| 43 | 1.133 | 9.289 | 9.049 | 0.391 | 0.289 |
| 44 | 1.066 | 7.073 | 5.985 | 0.396 | 0.293 |
| 45 | 1.108 | 5.715 | 3.986 | 0.508 | 0.297 |
| 46 | 1.054 | 5.64 | 4.902 | 0.491 | 0.229 |
| 47 | 0.531 | 5.951 | 4.819 | 0.308 | 0.633 |
| 48 | 1.695 | 7.293 | 5.583 | 0.459 | 0.094 |
| 49 | 0.911 | 5.72 | 4.389 | 0.366 | 0.265 |
| 50 | 1.253 | 6.876 | 5.873 | 0.576 | 0.152 |
| 51 | 0.362 | 7.316 | 5.227 | 0.329 | 1.204 |
| 52 | 1.076 | 6.715 | 5.389 | 0.137 | 0.249 |
| 53 | 0.594 | 6.381 | 6.906 | 0.157 | 0.668 |
| 54 | 0.526 | 6.345 | 5.668 | 0.231 | 0.574 |
| 55 | 0.637 | 6.387 | 5.165 | 0.126 | 0.451 |
| 56 | 0.546 | 7.182 | 5.91 | 0.141 | 0.582 |
| 57 | 0.811 | 7.285 | 5.476 | 0.158 | 0.417 |
| 58 | 0.681 | 7.28 | 6.839 | 0.09 | 0.397 |
| 59 | 1.936 | 5.394 | 4.582 | 0.655 | 0.105 |
| 60 | 1.806 | 6.316 | 5.687 | 0.548 | 0.048 |
| 61 | 1.938 | 7.963 | 7.09 | 0.349 | 0.177 |
| 62 | 1.655 | 7.035 | 5.215 | 0.407 | 0.092 |
| 63 | 1.638 | 7.552 | 6.047 | 0.664 | 0.103 |
| 64 | 0.54 | 6.224 | 5.474 | 0.224 | 0.587 |
| 65 | 0.43 | 6.548 | 6.984 | 0.061 | 0.682 |
| 66 | 1.709 | 8.282 | 7.877 | 0.385 | 0.32 |
| 67 | 0.595 | 8.662 | 5.132 | 0.169 | 0.678 |
| 68 | 0.604 | 6.415 | 4.844 | 0.207 | 0.525 |
| 69 | 1.03 | 4.706 | 3.408 | 0.314 | 0.309 |
| 70 | 0.846 | 6.931 | 6.065 | 0.145 | 0.299 |
| 71 | 0.96 | 7.7 | 6.133 | 0.163 | 0.381 |
| 72 | 2.538 | 7.316 | 6.206 | 0.946 | 0.101 |
| 73 | 0.633 | 5.669 | 4.595 | 0.476 | 0.503 |
| 74 | 0.952 | 5.024 | 5.039 | 0.444 | 0.362 |
| 75 | 0.815 | 6.546 | 6.052 | 0.08 | 0.435 |
| 76 | 1.901 | 7.582 | 6.972 | 0.683 | 0.121 |
| 77 | 1.207 | 5.133 | 3.936 | 0.129 | 0.199 |
| 78 | 0.953 | 6.397 | 5.002 | 0.27 | 0.248 |
| 79 | 2.375 | 6.961 | 6.537 | 0.475 | 0.257 |
| 80 | 0.79 | 5.672 | 4.828 | 0.143 | 0.346 |
| 81 | 0.519 | 5.982 | 4.755 | 0.105 | 0.565 |
| 82 | 0.497 | 6.802 | 5.907 | 0.189 | 0.816 |
| 83 | 0.812 | 5.328 | 4.464 | 0.426 | 0.355 |
| 84 | 1.021 | 5.942 | 4.679 | 0.452 | 0.248 |
| 85 | 0.796 | 8.219 | 7.99 | 0.255 | 0.42 |
| 86 | 2.404 | 7.903 | 7 | 0.275 | 0.135 |
| 87 | 1.292 | 7.293 | 6.152 | 0.49 | 0.21 |
| 88 | 0.642 | 6.558 | 5.21 | 0.237 | 0.579 |
| 89 | 0.97 | 7.665 | 6.672 | 0.242 | 0.287 |
| 90 | 1.14 | 7.583 | 6.796 | 0.22 | 0.319 |
| 91 | 0.765 | 8.109 | 6.796 | 0.098 | 0.364 |
| 92 | 2.296 | 11.454 | 8.101 | 0.096 | 0.143 |
| 93 | 1.376 | 7.458 | 6.076 | 0.409 | 0.218 |
| 94 | 1.092 | 11.584 | 8.18 | 0.174 | 0.229 |
| 95 | 1.44 | 8.841 | 8.494 | 0.356 | 0.325 |
| 96 | 2.729 | 8.936 | 7.981 | 0.395 | 0.215 |
| 97 | 0.454 | 9.637 | 9.004 | 0.119 | 0.85 |
| 98 | 0.714 | 4.024 | 3.263 | 0.262 | 0.466 |
| 99 | 0.41 | 6.186 | 5.295 | 0.063 | 0.86 |
| 100 | 0.32 | 5.283 | 4.744 | 0.134 | 1.073 |
| 101 | 0.875 | 5.459 | 4.287 | 0.219 | 0.304 |
| 102 | 0.707 | 6.873 | 5.441 | 0.082 | 0.395 |
| 103 | 2.144 | 9.042 | 7.879 | 0.122 | 0.071 |
| 104 | 1.424 | 5.302 | 4.127 | 0.414 | 0.11 |
| 105 | 0.855 | 6.817 | 5.46 | 0.154 | 0.397 |
| 106 | 0.766 | 6.157 | 4.832 | 0.17 | 0.4 |
| 107 | 0.611 | 6.348 | 5.75 | 0.209 | 0.965 |
| 108 | 0.751 | 7.634 | 6.204 | 0.103 | 0.509 |
| 109 | 1.372 | 8.666 | 7.966 | 0.066 | 0.103 |
| 110 | 1.451 | 6.318 | 5.513 | 0.139 | 0.087 |
| 111 | 1.504 | 7.3 | 6.645 | 0.16 | 0.205 |
| 112 | 1.075 | 6.422 | 5.529 | 0.1 | 0.228 |
| 113 | 0.618 | 6.066 | 5.701 | 0.073 | 0.505 |
| 114 | 0.616 | 7.535 | 6.498 | 0.057 | 0.496 |
| 115 | 1.363 | 8.734 | 7.134 | 0.184 | 0.202 |
| 116 | 0.566 | 5.791 | 4.259 | 0.068 | 0.592 |
| 117 | 1.93 | 4.705 | 4.314 | 0.58 | 0.1 |
| 118 | 0.822 | 6.66 | 4.933 | 0.126 | 0.342 |
| 119 | 0.708 | 7.826 | 6.912 | 0.093 | 0.529 |
| 120 | 1.235 | 5.967 | 5.758 | 0.459 | 0.464 |
| 121 | 0.747 | 4.058 | 4.903 | 0.272 | 0.356 |
| 122 | 0.523 | 5.824 | 6.779 | 0.307 | 0.535 |
| 123 | 1.02 | 3.75 | 5.104 | 0.464 | 0.345 |
| 124 | 0.926 | 2.685 | 4.107 | 0.627 | 0.308 |
| 125 | 0.734 | 5.304 | 7.248 | 0.287 | 0.371 |
| 126 | 1.311 | 3.44 | 4.937 | 0.457 | 0.286 |
| 127 | 0.772 | 3.626 | 4.943 | 0.279 | 0.366 |
| 128 | 0.408 | 3.222 | 4.211 | 0.302 | 0.698 |
| 129 | 0.517 | 3.123 | 4.19 | 0.418 | 0.82 |
| 130 | 1.216 | 4.311 | 6.028 | 0.619 | 0.27 |
| 131 | 0.964 | 4.57 | 5.911 | 0.255 | 0.341 |
| 132 | 0.63 | 4.687 | 6.141 | 0.354 | 0.644 |
| 133 | 0.479 | 1.795 | 2.493 | 0.259 | 0.573 |
| 134 | 1.02 | 4.719 | 5.932 | 0.462 | 0.303 |
| 135 | 0.914 | 2.663 | 3.869 | 0.53 | 0.384 |
| 136 | 0.561 | 4.806 | 6.427 | 0.299 | 0.504 |
| 137 | 0.981 | 4.127 | 4.501 | 0.616 | 0.276 |
| 138 | 1.167 | 2.868 | 4.042 | 0.896 | 0.268 |
| 139 | 1.647 | 5.842 | 7.158 | 0.453 | 0.148 |
| 140 | 0.541 | 4.773 | 5.749 | 0.215 | 0.579 |
| 141 | 1.048 | 5.455 | 6.199 | 0.268 | 0.285 |
| 142 | 2.149 | 3.329 | 3.645 | 1.18 | 0.125 |
| 143 | 1.149 | 5.286 | 6.546 | 0.379 | 0.222 |
| 144 | 0.834 | 6.598 | 7.584 | 0.279 | 0.337 |
| 145 | 1.034 | 2.834 | 3.635 | 0.431 | 0.235 |
| 146 | 0.989 | 5.05 | 5.726 | 0.196 | 0.271 |
| 147 | 0.952 | 3.457 | 3.651 | 0.434 | 0.442 |
| 148 | 0.868 | 4.644 | 4.497 | 0.518 | 0.392 |
| 149 | 1.237 | 3.228 | 3.532 | 0.526 | 0.132 |
| 150 | 0.981 | 5.032 | 5.596 | 0.228 | 0.274 |
| 151 | 1.617 | 4.031 | 4.124 | 0.605 | 0.189 |
| 152 | 0.567 | 3.69 | 3.74 | 0.114 | 0.638 |
| 153 | 2.035 | 3.828 | 3.541 | 0.837 | 0.169 |
| 154 | 1.778 | 3.937 | 3.647 | 0.854 | 0.426 |
| 155 | 0.499 | 5.65 | 5.359 | 0.076 | 0.581 |
| 156 | 1.383 | 5.944 | 5.567 | 0.23 | 0.092 |
| 157 | 1.962 | 4.708 | 4.399 | 0.598 | 0.045 |
| 158 | 1.141 | 4.848 | 4.426 | 0.165 | 0.156 |
| 159 | 1.88 | 4.109 | 3.668 | 0.501 | 0.077 |
| 160 | 1.147 | 6.11 | 5.61 | 0.176 | 0.171 |
| 161 | 1.522 | 4.418 | 3.958 | 0.43 | 0.072 |
| 162 | 1.231 | 3.646 | 3.285 | 0.405 | 0.177 |
| 163 | 1.049 | 3.654 | 3.914 | 0.272 | 0.192 |
| 164 | 1.038 | 2.94 | 2.496 | 0.302 | 0.387 |
| 165 | 1.737 | 6.579 | 6.208 | 0.521 | 0.085 |
| 166 | 1.338 | 3.167 | 3.202 | 0.342 | 0.116 |
| 167 | 0.826 | 5.243 | 4.374 | 0.097 | 0.486 |
| 168 | 1.159 | 3.151 | 2.731 | 0.572 | 0.154 |
| 169 | 0.801 | 2.909 | 2.481 | 0.21 | 0.341 |
| 170 | 0.544 | 3.541 | 3.109 | 0.157 | 0.535 |
| 171 | 0.974 | 3.509 | 3.701 | 0.139 | 0.335 |
| 172 | 1.17 | 4.8 | 4.926 | 0.177 | 0.18 |
| 173 | 1.178 | 7.32 | 7.009 | 0.099 | 0.205 |
| 174 | 2.118 | 7.236 | 6.963 | 0.231 | 0.146 |
| 175 | 0.679 | 4.174 | 3.5 | 0.048 | 0.445 |
| 176 | 0.749 | 4.704 | 4.63 | 0.131 | 0.364 |
| 177 | 2.197 | 5.853 | 5.263 | 0.419 | 0.031 |
| 178 | 1.588 | 3.808 | 3.525 | 0.458 | 0.059 |
| 179 | 0.893 | 3.436 | 3.011 | 0.071 | 0.276 |
| 180 | 0.845 | 2.932 | 3.059 | 0.163 | 0.337 |
| 181 | 1.522 | 7.239 | 6.868 | 0.2 | 0.084 |
| 182 | 1.221 | 6.428 | 5.859 | 0.232 | 0.175 |
| 183 | 0.996 | 3.451 | 3.522 | 0.11 | 0.246 |
| 184 | 0.66 | 5.406 | 5.286 | 0.04 | 0.419 |
| 185 | 0.886 | 4.224 | 3.614 | 0.172 | 0.257 |
| 186 | 0.982 | 5.14 | 4.906 | 0.074 | 0.263 |
| 187 | 1.539 | 3.99 | 3.6 | 0.375 | 0.07 |
| 188 | 1.132 | 5.873 | 5.472 | 0.161 | 0.164 |
| 189 | 0.99 | 3.004 | 2.716 | 0.238 | 0.206 |
| 190 | 0.919 | 3.733 | 3.477 | 0.051 | 0.318 |
| 191 | 0.715 | 6.324 | 6.187 | 0.075 | 0.497 |
| 192 | 1.192 | 4.234 | 4.163 | 0.141 | 0.163 |
| 193 | 0.969 | 5.876 | 5.41 | 0.066 | 0.251 |
| 194 | 1.22 | 3.295 | 2.863 | 0.177 | 0.164 |
| 195 | 1.497 | 3.727 | 3.41 | 0.183 | 0.07 |
| 196 | 1.244 | 4.027 | 3.105 | 0.204 | 0.12 |
| 197 | 1.361 | 4.544 | 4.472 | 0.197 | 0.098 |
| 198 | 1.385 | 7.236 | 6.464 | 0.064 | 0.111 |
| 199 | 1.503 | 5.891 | 5.179 | 0.083 | 0.096 |
| 200 | 1.232 | 4.267 | 4.111 | 0.131 | 0.125 |

Source: The author recorded the epoch data of the machine learning program when training the model.
