# Supplementary material for: Artificial intelligence empowering museum space layout design: Insights from China
Source: PLoS One. 2024 Nov 7;19(11):e0310594. doi: 10.1371/journal.pone.0310594 (PMC11542801; doi:10.1371/journal.pone.0310594)
Supplement: S6 File — (DOCX) [file pone.0310594.s006.docx]

# S6. Numerical Statistics During the Model 2 Epoch Period

In the Epoch cycle of Model 2, the detailed numerical statistics of G_GAN, G_GAN_Feat, G_VGG, D_real, and D_fake are as follows:

| epoch | G_GAN | G_GAN_Feat | G_VGG | D_real | D_fake |
| --- | --- | --- | --- | --- | --- |
| 1 | 0.774 | 2.549 | 3.42 | 0.672 | 0.557 |
| 2 | 0.607 | 3.32 | 4.692 | 0.621 | 0.543 |
| 3 | 0.49 | 2.148 | 4.829 | 0.425 | 0.607 |
| 4 | 0.649 | 3.04 | 3.903 | 0.678 | 0.441 |
| 5 | 0.773 | 2.076 | 4.411 | 0.61 | 0.403 |
| 6 | 0.679 | 2.764 | 6.137 | 0.564 | 0.514 |
| 7 | 0.928 | 3.922 | 4.132 | 0.901 | 0.326 |
| 8 | 0.699 | 2.186 | 4.446 | 0.657 | 0.539 |
| 9 | 1.004 | 2.826 | 4.391 | 0.57 | 0.245 |
| 10 | 0.838 | 3.008 | 3.263 | 0.437 | 0.375 |
| 11 | 0.486 | 3.142 | 5.879 | 0.337 | 0.736 |
| 12 | 0.46 | 2.844 | 4.219 | 0.305 | 0.718 |
| 13 | 0.447 | 2.076 | 2.539 | 0.437 | 0.635 |
| 14 | 0.805 | 2.582 | 2.95 | 0.321 | 0.34 |
| 15 | 1.749 | 2.662 | 3.613 | 1.291 | 0.083 |
| 16 | 1.133 | 3.894 | 4.244 | 0.248 | 0.163 |
| 17 | 0.6 | 2.85 | 3.142 | 0.622 | 0.561 |
| 18 | 1.001 | 3.391 | 3.257 | 0.573 | 0.326 |
| 19 | 0.59 | 5.139 | 5.692 | 0.45 | 0.906 |
| 20 | 0.806 | 2.839 | 3.729 | 0.742 | 0.433 |
| 21 | 0.314 | 4.972 | 4.303 | 0.244 | 0.879 |
| 22 | 0.907 | 3.209 | 3.465 | 0.646 | 0.34 |
| 23 | 0.875 | 5.073 | 4.268 | 0.286 | 0.333 |
| 24 | 0.486 | 1.674 | 2.513 | 0.709 | 0.594 |
| 25 | 0.866 | 3.213 | 3.435 | 0.444 | 0.336 |
| 26 | 0.675 | 5.358 | 4.009 | 0.19 | 0.452 |
| 27 | 0.787 | 3.97 | 3.196 | 0.369 | 0.387 |
| 28 | 0.548 | 2.581 | 3.373 | 0.491 | 0.787 |
| 29 | 0.469 | 3.379 | 3.158 | 0.113 | 0.689 |
| 30 | 0.807 | 6.357 | 4.299 | 0.233 | 0.428 |
| 31 | 0.594 | 3.221 | 3.768 | 0.357 | 0.46 |
| 32 | 0.522 | 2.892 | 3.69 | 0.313 | 0.525 |
| 33 | 0.684 | 1.954 | 4.06 | 0.585 | 0.396 |
| 34 | 0.725 | 2.941 | 3.659 | 0.558 | 0.541 |
| 35 | 1.058 | 2.688 | 2.898 | 0.78 | 0.245 |
| 36 | 1.017 | 3.628 | 4.723 | 0.675 | 0.343 |
| 37 | 0.971 | 3.171 | 3.486 | 0.608 | 0.51 |
| 38 | 1.035 | 2.959 | 4.162 | 0.78 | 0.266 |
| 39 | 0.357 | 2.143 | 2.104 | 0.342 | 0.751 |
| 40 | 0.392 | 2.652 | 3.114 | 0.287 | 0.842 |
| 41 | 0.46 | 2.094 | 2.954 | 0.423 | 0.683 |
| 42 | 0.476 | 2.311 | 3.199 | 0.264 | 0.663 |
| 43 | 0.485 | 1.42 | 2.298 | 0.426 | 0.602 |
| 44 | 0.551 | 3.371 | 3.229 | 0.387 | 0.532 |
| 45 | 0.688 | 1.928 | 2.924 | 0.63 | 0.397 |
| 46 | 0.5 | 2.803 | 4.201 | 0.457 | 0.709 |
| 47 | 0.414 | 2.156 | 2.633 | 0.328 | 0.68 |
| 48 | 0.67 | 1.272 | 2.227 | 0.644 | 0.447 |
| 49 | 0.218 | 2.563 | 2.509 | 0.166 | 1.143 |
| 50 | 0.392 | 1.88 | 2.346 | 0.3 | 0.669 |
| 51 | 0.548 | 2.189 | 2.447 | 0.324 | 0.52 |
| 52 | 0.741 | 1.557 | 1.939 | 0.621 | 0.38 |
| 53 | 0.804 | 2.578 | 2.808 | 0.576 | 0.342 |
| 54 | 0.777 | 1.298 | 2.377 | 0.674 | 0.348 |
| 55 | 0.434 | 3.555 | 3.372 | 0.352 | 0.711 |
| 56 | 0.577 | 1.358 | 2.125 | 0.508 | 0.563 |
| 57 | 0.801 | 2.394 | 2.823 | 0.597 | 0.465 |
| 58 | 0.464 | 3.085 | 3.758 | 0.274 | 0.696 |
| 59 | 0.819 | 1.025 | 1.462 | 0.831 | 0.332 |
| 60 | 1.457 | 3.489 | 3.843 | 0.686 | 0.427 |
| 61 | 0.703 | 1.753 | 1.878 | 0.56 | 0.417 |
| 62 | 0.543 | 9.696 | 7.526 | 0.111 | 0.516 |
| 63 | 0.882 | 1.674 | 2.445 | 0.779 | 0.303 |
| 64 | 1.046 | 1.646 | 2.442 | 0.956 | 0.216 |
| 65 | 0.534 | 1.822 | 2.057 | 0.411 | 0.489 |
| 66 | 0.567 | 0.779 | 1.342 | 0.519 | 0.536 |
| 67 | 0.571 | 1.967 | 2.324 | 0.359 | 0.478 |
| 68 | 0.504 | 1.932 | 2.421 | 0.366 | 0.758 |
| 69 | 0.472 | 1.845 | 2.216 | 0.324 | 0.61 |
| 70 | 0.386 | 1.521 | 2.487 | 0.33 | 0.703 |
| 71 | 0.613 | 1.521 | 1.879 | 0.488 | 0.606 |
| 72 | 0.463 | 2.643 | 3.455 | 0.384 | 0.662 |
| 73 | 0.533 | 4.35 | 5.245 | 0.332 | 0.674 |
| 74 | 0.537 | 2.154 | 1.815 | 0.329 | 0.58 |
| 75 | 0.639 | 0.782 | 1.218 | 0.602 | 0.443 |
| 76 | 0.691 | 1.669 | 1.645 | 0.506 | 0.648 |
| 77 | 0.573 | 0.988 | 1.431 | 0.53 | 0.471 |
| 78 | 0.557 | 1.734 | 2.658 | 0.532 | 0.543 |
| 79 | 0.841 | 1.309 | 1.898 | 0.699 | 0.347 |
| 80 | 0.408 | 1.238 | 1.627 | 0.335 | 0.792 |
| 81 | 0.688 | 1.842 | 1.836 | 0.543 | 0.38 |
| 82 | 0.512 | 1.321 | 2.259 | 0.47 | 0.547 |
| 83 | 0.963 | 1.369 | 1.408 | 0.851 | 0.22 |
| 84 | 0.68 | 1.239 | 1.881 | 0.613 | 0.397 |
| 85 | 0.441 | 7.434 | 6.773 | 0.177 | 0.739 |
| 86 | 1.093 | 1.721 | 2.559 | 1.019 | 0.284 |
| 87 | 0.911 | 0.987 | 1.332 | 0.813 | 0.342 |
| 88 | 0.837 | 1.536 | 1.56 | 0.705 | 0.335 |
| 89 | 0.389 | 1.557 | 1.74 | 0.346 | 0.829 |
| 90 | 0.441 | 1.905 | 2.165 | 0.312 | 0.602 |
| 91 | 0.436 | 1.287 | 1.551 | 0.369 | 0.628 |
| 92 | 0.585 | 1.133 | 1.596 | 0.528 | 0.498 |
| 93 | 0.638 | 7.018 | 1.496 | 0.108 | 0.447 |
| 94 | 0.352 | 1.047 | 1.335 | 0.296 | 0.759 |
| 95 | 1.058 | 1.521 | 1.394 | 0.832 | 0.3 |
| 96 | 0.802 | 0.762 | 1.041 | 0.73 | 0.331 |
| 97 | 0.835 | 7.481 | 1.416 | 0.141 | 0.366 |
| 98 | 0.429 | 0.734 | 1.004 | 0.389 | 0.655 |
| 99 | 0.705 | 1.214 | 1.21 | 0.64 | 0.427 |
| 100 | 0.863 | 0.958 | 1.251 | 0.787 | 0.339 |
| 101 | 0.554 | 1.554 | 1.861 | 0.42 | 0.473 |
| 102 | 1.02 | 1.003 | 1.269 | 0.98 | 0.234 |
| 103 | 0.436 | 1.073 | 1.493 | 0.409 | 1.025 |
| 104 | 0.365 | 1.405 | 1.395 | 0.296 | 0.756 |
| 105 | 0.663 | 1.826 | 1.912 | 0.534 | 0.472 |
| 106 | 0.668 | 1.327 | 1.755 | 0.55 | 0.397 |
| 107 | 0.57 | 1.32 | 1.246 | 0.367 | 0.457 |
| 108 | 0.569 | 1.865 | 2.112 | 0.452 | 0.588 |
| 109 | 0.521 | 1.363 | 1.491 | 0.416 | 0.526 |
| 110 | 0.285 | 1.791 | 1.91 | 0.22 | 0.855 |
| 111 | 0.88 | 1.577 | 1.545 | 0.578 | 0.279 |
| 112 | 0.427 | 0.29 | 0.882 | 0.425 | 0.599 |
| 113 | 0.557 | 0.523 | 1.127 | 0.546 | 0.48 |
| 114 | 0.611 | 0.92 | 1.391 | 0.591 | 0.424 |
| 115 | 0.497 | 0.743 | 2.06 | 0.491 | 0.52 |
| 116 | 0.538 | 0.44 | 1.309 | 0.533 | 0.484 |
| 117 | 0.646 | 1.304 | 2.287 | 0.595 | 0.382 |
| 118 | 0.33 | 0.734 | 1.552 | 0.323 | 0.884 |
| 119 | 0.515 | 0.838 | 1.495 | 0.496 | 0.498 |
| 120 | 0.4 | 1.121 | 1.914 | 0.393 | 0.813 |
| 121 | 0.639 | 0.797 | 1.492 | 0.608 | 0.442 |
| 122 | 0.478 | 1.08 | 1.243 | 0.417 | 0.615 |
| 123 | 0.289 | 0.921 | 1.131 | 0.286 | 0.917 |
| 124 | 0.512 | 0.925 | 1.219 | 0.491 | 0.556 |
| 125 | 0.596 | 3.304 | 3.28 | 0.472 | 0.616 |
| 126 | 0.582 | 0.406 | 0.739 | 0.583 | 0.471 |
| 127 | 0.424 | 1.032 | 2.097 | 0.414 | 0.622 |
| 128 | 0.415 | 0.443 | 0.983 | 0.412 | 0.617 |
| 129 | 0.617 | 0.541 | 1.284 | 0.609 | 0.548 |
| 130 | 0.538 | 0.575 | 1.125 | 0.528 | 0.496 |
| 131 | 0.602 | 0.656 | 1.332 | 0.588 | 0.46 |
| 132 | 0.757 | 0.504 | 1.211 | 0.732 | 0.335 |
| 133 | 0.57 | 2.711 | 4.715 | 0.451 | 0.483 |
| 134 | 0.711 | 0.648 | 0.684 | 0.705 | 0.356 |
| 135 | 0.538 | 0.837 | 1.318 | 0.509 | 0.52 |
| 136 | 0.535 | 0.84 | 1.057 | 0.48 | 0.497 |
| 137 | 0.782 | 1.039 | 1.409 | 0.677 | 0.368 |
| 138 | 0.68 | 0.797 | 1.518 | 0.659 | 0.41 |
| 139 | 0.471 | 0.909 | 1.482 | 0.435 | 0.574 |
| 140 | 0.553 | 1.32 | 1.463 | 0.501 | 0.491 |
| 141 | 0.63 | 0.838 | 1.095 | 0.579 | 0.454 |
| 142 | 0.544 | 0.925 | 1.338 | 0.518 | 0.486 |
| 143 | 0.523 | 1.549 | 1.229 | 0.407 | 0.506 |
| 144 | 0.585 | 1.397 | 1.263 | 0.455 | 0.455 |
| 145 | 0.535 | 0.926 | 0.923 | 0.511 | 0.489 |
| 146 | 0.454 | 2.049 | 2.01 | 0.363 | 0.566 |
| 147 | 0.617 | 0.977 | 0.946 | 0.552 | 0.44 |
| 148 | 0.525 | 1.066 | 1.088 | 0.442 | 0.509 |
| 149 | 0.565 | 0.658 | 0.859 | 0.515 | 0.471 |
| 150 | 0.478 | 0.672 | 0.936 | 0.438 | 0.554 |
| 151 | 0.489 | 1.188 | 1.088 | 0.413 | 0.531 |
| 152 | 0.51 | 1.205 | 1.445 | 0.444 | 0.535 |
| 153 | 1.021 | 0.941 | 0.855 | 0.976 | 0.271 |
| 154 | 0.625 | 1.255 | 1.286 | 0.574 | 0.488 |
| 155 | 0.568 | 0.676 | 0.793 | 0.543 | 0.472 |
| 156 | 0.693 | 0.878 | 1.073 | 0.594 | 0.361 |
| 157 | 0.582 | 1.015 | 1.152 | 0.498 | 0.561 |
| 158 | 0.498 | 1.519 | 1.426 | 0.37 | 0.512 |
| 159 | 0.316 | 2.114 | 1.693 | 0.243 | 0.802 |
| 160 | 0.443 | 2.186 | 1.92 | 0.317 | 0.604 |
| 161 | 0.336 | 1.288 | 1.152 | 0.301 | 0.767 |
| 162 | 0.663 | 0.718 | 0.777 | 0.618 | 0.394 |
| 163 | 0.665 | 0.962 | 0.998 | 0.593 | 0.393 |
| 164 | 0.717 | 1.248 | 1.274 | 0.64 | 0.354 |
| 165 | 0.517 | 0.594 | 0.656 | 0.491 | 0.575 |
| 166 | 0.428 | 1.236 | 1.032 | 0.379 | 0.596 |
| 167 | 0.555 | 1.005 | 1.144 | 0.445 | 0.477 |
| 168 | 0.425 | 1.168 | 1.003 | 0.37 | 0.618 |
| 169 | 0.477 | 2.931 | 2.263 | 0.329 | 0.543 |
| 170 | 0.46 | 0.736 | 0.928 | 0.395 | 0.562 |
| 171 | 0.443 | 0.728 | 0.899 | 0.379 | 0.578 |
| 172 | 0.783 | 0.782 | 0.84 | 0.725 | 0.296 |
| 173 | 0.592 | 1.226 | 0.816 | 0.503 | 0.441 |
| 174 | 0.691 | 1.405 | 1.078 | 0.59 | 0.366 |
| 175 | 0.735 | 1.123 | 1.013 | 0.626 | 0.37 |
| 176 | 0.479 | 0.715 | 0.829 | 0.453 | 0.539 |
| 177 | 0.488 | 0.701 | 0.454 | 0.449 | 0.53 |
| 178 | 0.367 | 1.685 | 1.472 | 0.274 | 0.67 |
| 179 | 0.507 | 1.111 | 0.727 | 0.411 | 0.506 |
| 180 | 0.577 | 0.892 | 0.867 | 0.517 | 0.438 |
| 181 | 0.451 | 1.535 | 1.53 | 0.395 | 0.585 |
| 182 | 0.548 | 0.969 | 0.984 | 0.45 | 0.477 |
| 183 | 0.833 | 6.475 | 1.154 | 0.179 | 0.292 |
| 184 | 0.877 | 0.607 | 0.511 | 0.854 | 0.246 |
| 185 | 0.691 | 0.86 | 0.888 | 0.513 | 0.394 |
| 186 | 0.441 | 0.638 | 0.49 | 0.414 | 0.583 |
| 187 | 0.452 | 0.874 | 0.853 | 0.355 | 0.562 |
| 188 | 0.39 | 1.08 | 0.949 | 0.346 | 0.665 |
| 189 | 0.596 | 0.738 | 0.677 | 0.514 | 0.427 |
| 190 | 0.456 | 1.178 | 0.782 | 0.394 | 0.56 |
| 191 | 0.433 | 0.87 | 0.771 | 0.349 | 0.588 |
| 192 | 0.609 | 0.82 | 0.84 | 0.492 | 0.438 |
| 193 | 0.664 | 1.126 | 0.813 | 0.539 | 0.379 |
| 194 | 0.587 | 0.615 | 0.441 | 0.548 | 0.43 |
| 195 | 0.511 | 0.664 | 0.519 | 0.478 | 0.5 |
| 196 | 0.59 | 0.485 | 0.418 | 0.532 | 0.429 |
| 197 | 0.576 | 1.902 | 1.214 | 0.381 | 0.45 |
| 198 | 0.515 | 1.283 | 1.446 | 0.428 | 0.496 |
| 199 | 0.543 | 0.72 | 0.618 | 0.476 | 0.466 |
| 200 | 0.533 | 1.137 | 0.695 | 0.51 | 0.477 |

Source: The author recorded the epoch data of the machine learning program when training the model.
