# Supplementary material for: Artificial intelligence empowering museum space layout design: Insights from China
Source: PLoS One. 2024 Nov 7;19(11):e0310594. doi: 10.1371/journal.pone.0310594 (PMC11542801; doi:10.1371/journal.pone.0310594)
Supplement: S7 File — (DOCX) [file pone.0310594.s007.docx]

# S7. Museum Space Layout Design Satisfaction Questionnaire

Dear Sirs and Ladies, in order to investigate the public’s satisfaction with the spatial layout of museums, we have made two different layout designs for museums with different floor plans. Please rate these plans according to your preferences or professional abilities. Thank you for your support and cooperation. The possible answers to the questions are 1, 2, 3, 4, and 5. Among them, 5 means that you strongly agree with the plan (very satisfied, very good), and 4 means that you agree somewhat (relatively satisfied, relatively good). 3 represents general agreement (okay), 2 represents relative disapproval (not very good, not very satisfied), and 1 represents disapproval (not good, not satisfied). Please choose according to your true opinion and knowledge and mark your desired score with √. By clicking the next button and completing the survey you indicate that you have consented to participate in this research. If you do not want to participate, please close the browser.

| **Group 1: Comparison of Square Museum Floor Plan Designs** | | | | | |
| --- | --- | --- | --- | --- | --- |
| Design plan (1) | 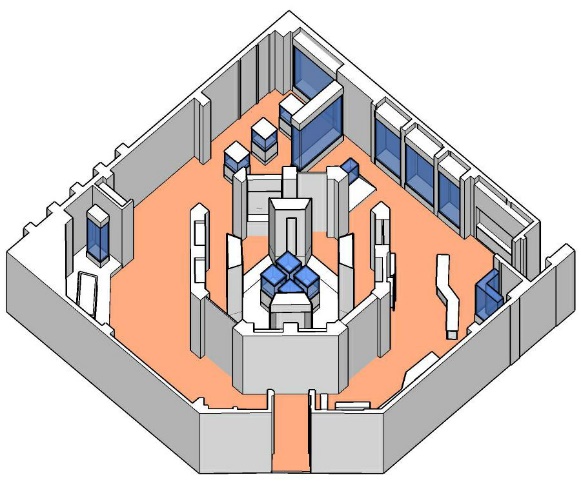 | | | | |
| Evaluate satisfaction | 5 | 4 | 3 | 2 | 1 |
| Design plan (2) | 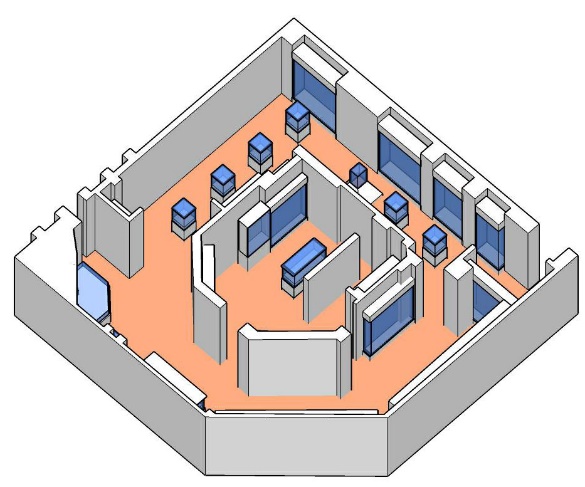 | | | | |
| Evaluate satisfaction | 5 | 4 | 3 | 2 | 1 |
| **Group 2: Comparison of “T” Shaped Museum Floor Plan Designs** | | | | | |
| Design plan (1) | 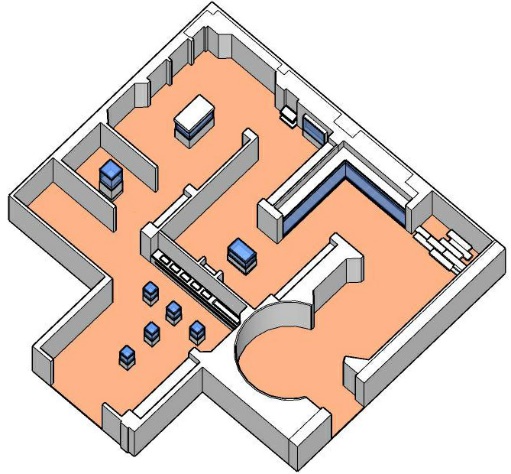 | | | | |
| Evaluate satisfaction | 5 | 4 | 3 | 2 | 1 |
| Design plan (2) | 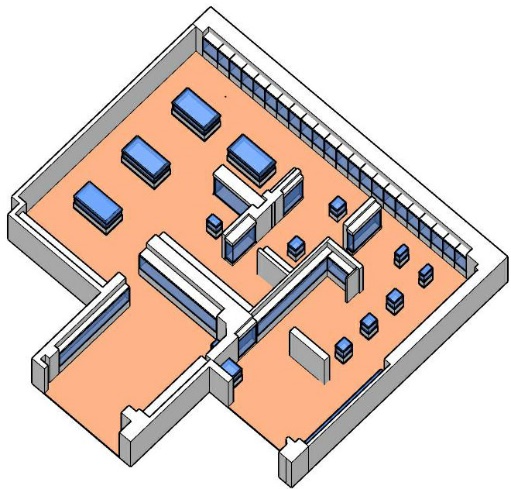 | | | | |
| Evaluate satisfaction | 5 | 4 | 3 | 2 | 1 |
| **Group 3: Comparison of Oval Museum Floor Plan Designs** | | | | | |
| Design plan (1) | 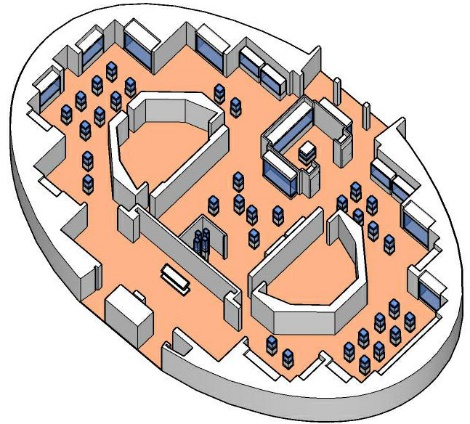 | | | | |
| Evaluate satisfaction | 5 | 4 | 3 | 2 | 1 |
| Design plan (2) | 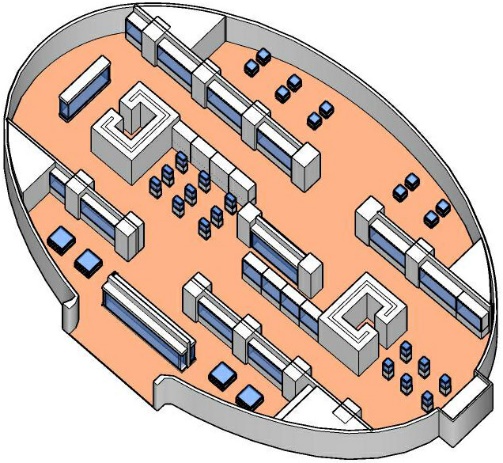 | | | | |
| Evaluate satisfaction | 5 | 4 | 3 | 2 | 1 |
| **Group 4: Comparison of Rectangular Museum Floor Plan Designs** | | | | | |
| Design plan (1) | 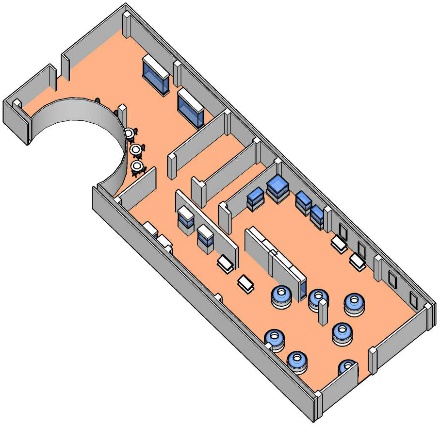 | | | | |
| Evaluate satisfaction | 5 | 4 | 3 | 2 | 1 |
| Design plan (2) | 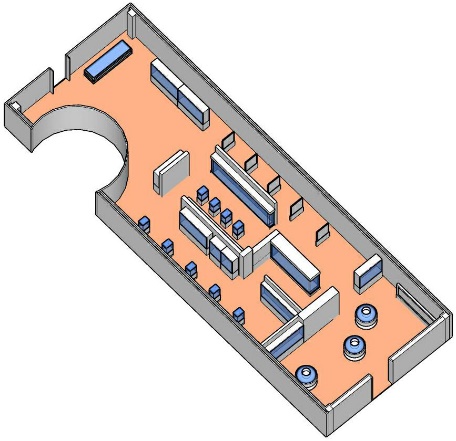 | | | | |
| Evaluate satisfaction | 5 | 4 | 3 | 2 | 1 |
| **Group 5: Comparison of Double-Arc Museum Floor Plan Designs** | | | | | |
| Design plan (1) | 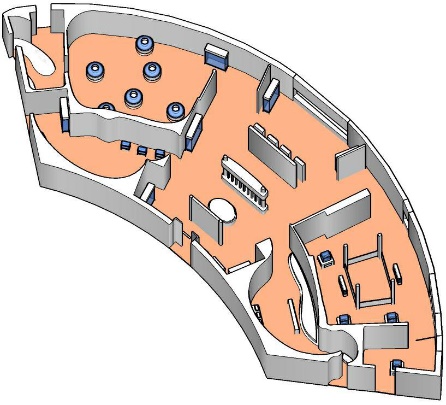 | | | | |
| Evaluate satisfaction | 5 | 4 | 3 | 2 | 1 |
| Design plan (2) | 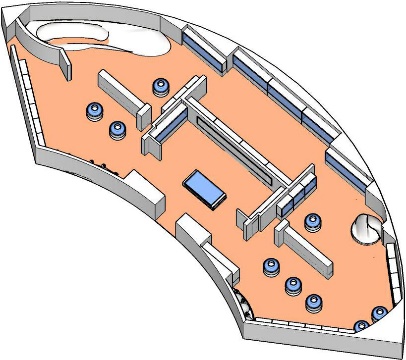 | | | | |
| Evaluate satisfaction | 5 | 4 | 3 | 2 | 1 |

Thank you again for your support! We promise that the personal information given in the questionnaire will be kept strictly confidential and anonymous. The researchers will only extract the final satisfaction score and use it strictly for this academic research.
